# Metal ureteral stents for ureteral stricture: 2 years of experience with 246 cases

**DOI:** 10.1097/JS9.0000000000000841

**Published:** 2023-10-06

**Authors:** Xiaoshuai Gao, Xingpeng Di, Guo Chen, Wei Wang, Liao Peng, Jixiang Chen, Xin Wei

**Affiliations:** Department of Urology and Institute of Urology (Laboratory of Reconstructive Urology), West China Hospital, Sichuan University, Chengdu, Sichuan, People’s Republic of China

**Keywords:** kidney transplantation, metal stents, recurrent strictures, ureteral stricture

## Abstract

**Background:**

Metal ureteral stents (MUS) has gained popularity as an endoscopic treatment alternative for the management of ureteral strictures. The aim of this study was to evaluate the safety, efficacy, and tolerability of MUS for treating ureteral strictures and to identify any factors that could influence the success of this intervention.

**Methods:**

This study is a prospective analysis of the efficacy and safety of MUS for treating ureteral strictures in a single-center setting. The study enrolled 246 patients who had been diagnosed with ureteral strictures and had undergone MUS placement between January 2019 and July 2021. The patients were followed-up for a duration of 2 years.

**Results:**

The overall success rate of MUS placement was 71.7%. Furthermore, the success rate of ureteral strictures after kidney transplantation (78.2%) was significantly higher than common ureteral strictures (73.0%) or recurrent ureteral strictures (67.6%). Additionally, postsurgery, there was a considerable reduction in hydronephrosis volume (68.9±96.1 vs. 32.1±48.8 cm^3^), blood creatinine level (103.7±49.8 vs. 94.4±47.5 mol/l) and urea nitrogen level (6.7±7.2 vs. 5.1±2.4 mmol/l). The study also reported that the rate of adverse events associated with MUS was relatively low, included hematuria (7.9%), pain (6.8%), urinary tract infection (6.4%), and lower urinary tract symptoms (5.3%).

**Conclusions:**

MUS appear to be a safe and effective treatment option for ureteral strictures, with a high success rate and low complication rate. These results have important implications for the management of ureteral strictures and can help guide clinical decision-making in the selection of treatment options.

## Introduction

Highlights

- Metal ureteral stents is safe and effective for ureteral strictures.
- Metal ureteral stents offers a novel option for the treatment of strictures.
- These endoscopic techniques can be selected for suitable patients.

Ureteral strictures can be caused by a variety of factors, including urinary system stones, previous surgeries, radiation therapy, and chronic inflammatory conditions^1^. The symptoms of ureteral strictures can range from mild discomfort to severe renal failure in cases of complete obstruction. Traditional ureteroplasty can achieve satisfactory results but is associated with high rates of complications, such as bleeding, perforation, and stricture recurrence^2^. Additionally, ureteroplasty surgery requires a longer hospital stay and recovery time. Moreover, the management of recurrent ureteral strictures after ureteroplasty or kidney transplantation with open surgery is highly challenging and associated with significant morbidity^3,4^. Endoscopic treatment options, including balloon dilation, ureteroscopic incision, and stenting, have become increasingly popular for the management of ureteral strictures due to their minimally invasive nature and high success rates^5,6^.

In recent years, the use of Allium metal ureteral stents (MUS) has gained popularity as a endoscopic treatment alternative for the management of ureteral strictures^7^. MUS offer several advantages over traditional techniques, including a lower risk of complications and a higher success rate in preventing stricture recurrence^8^. In addition, MUS have emerged as a promising treatment option for recurrent ureteral strictures due to their durability and long-term patency rates^9^.

The purpose of this study is to present our experience with the use of MUS for the management of ureteral strictures. We report on a series of 246 cases over a 2-year period, highlighting the outcomes and complications associated with this treatment approach. Through our experience, we aim to evaluate the efficacy and safety of MUS as a treatment option for ureteral strictures and provide insights into the optimal patient selection, MUS placement techniques, and follow-up strategies. Our findings may help guide clinical decision-making and improve patient outcomes in the management of this challenging urological condition.

## Methods

### Study design

This was a prospective, single-center study of patients who underwent Allium MUS placement for ureteral strictures between January 2019 and July 2021. This study was reported in line with the strengthening the reporting of cohort, cross-sectional, and case–control studies in surgery (STROCSS) criteria^10^.

### Patient selection

The inclusion criteria for our study consisted of patients clinically diagnosed with ureteral stricture, aged 14 years or older. Prior to the operation, our patients underwent either computed tomography urography or retrograde ureterography to precisely identify the location and length of the ureteral stricture. Additionally, renograms or single-photon emission CT (SPECT) scans were performed to assess the presence of obstruction and evaluate the renal function of the affected kidney. These diagnostic procedures were employed to gather comprehensive information before proceeding with the surgery. On the other hand, the following criteria were used for exclusion: (1) Uncontrolled acute or chronic inflammation of the genitourinary system. (2) Presence of severe hematuria, which may impede visualization during endoscopy. (3) Severe urethral stricture that hinders endoscope insertion and surgical procedures. (4) Pregnancy or menstruation in female patients. (5) Patients with severe systemic diseases who are unable to tolerate anesthesia or surgery.

### Data collection

Patient data were collected from electronic medical records including sex, age, height, weight, BMI, etiology and length of stricture, urea nitrogen levels, serum creatinine, hydronephrosis volume, operative time, symptoms with MUS, length of hospital stay, and hospital costs. The hydronephrosis volume was calculated by abdominal computed tomography (CT): hydronephrosis volume=length×width×depth×0.523^11^.

### MUS placement

MUS placement was performed under general anesthesia with the patient in the lithotomy position. The location and length of the ureteral stricture were determined using retrograde or anterograde radiography under fluoroscopic guidance. To perform the procedure, a rigid cystoscope was carefully inserted into the bladder. Subsequently, a guidewire was inserted retrogradely into the obstructed ureter under precise guidance. Then, a ureteral balloon dilation catheter was inserted at the site of obstruction, and the stricture was dilated up to 25 atmospheres for a duration of 3 min. Once it was confirmed that the narrowed segment had been adequately dilated under fluoroscopy, a MUS delivery system was inserted with a guidewire. The MUS was then deployed within the narrowed portion of the ureter under fluoroscopy. Following successful stent placement, radiography was performed again to verify the position of the stent and the patency of the ureter. The choice of MUS length, either 10 cm or 12 cm, depended on the length of the ureteral stricture. It was ensured that the MUS support extended at least 2 cm beyond both ends of the narrowed segment. In cases of ureteral strictures after kidney transplantation, all patients have undergone a nephrostomy to protect renal function. A guidewire was introduced via a nephrostomy and passed through the narrow segment into the bladder. The guidewire was then pulled out through the urethra using a cystoscope. Subsequently, a ureteral balloon was retrogradely inserted along the guidewire and through the stricture. The balloon dilator was inflated to 20 atm (1.72 MPa) for 3 min to create adequate space for the insertion of a 10 cm MUS. A 6-Fr flexible ureteroscope sheath and a second working wire were then retrogradely inserted above the guidewire into the renal pelvis. Finally, the MUS was accurately positioned with the aid of the sheath. When necessary, the MUS could be easily disassembled and removed by pulling its end under ureteroscopy^7^.

### Follow-up

All patients were followed-up regularly with abdominal CT, serum creatinine, and urea nitrogen every 3 months after surgery. All patients had a follow-up period of 2 years. The need for additional interventions, such as repeat MUS placement, was also recorded. Surgical success was defined as the absence of any need to replace the MUS due to migration, occlusion, or encrustation.

### Statistical analysis

Descriptive statistics were used to summarize patient demographics and clinical characteristics. Continuous variables were presented as mean±SD, while categorical variables were described as frequency (percentage). A paired *t*-test was used to compare continuous variables before and after surgery. All statistical analyses were performed using SPSS software version 22.0, and a *P*-value <0.05 was considered statistically significant.

## Results

The general characteristics are summarized in Table 1. Two hundred and forty-six patients with 265 renal units underwent MUS insertion, of which 19 patients had bilateral ureteral strictures. The mean age was 46.5 years, and 64.2% of patients were male. The most common etiology of stricture was ureteroplasty (40.8%), followed by urinary stones (29.1%), kidney transplantation (17.4%), abdominal and pelvic surgery or radiotherapy (7.5%), and urinary cancer (5.3%). The most frequent location of the stricture was the proximal ureter (44.9%), followed by the ureterovesical anastomosis (24.5%), distal ureter (19.2%), and the middle ureter (11.3%). The average length of ureteral strictures in our study was (3.4±3.0) cm.

**Table 1:** General characteristics of the patients.

Variable	Overall
Number of patients, *n*	246
Number of renal units, *n*	265
Age, years	46.5±14.2
Sex, male/female, *n*	158/88
BMI, kg/m^2^	23.8±3.2
Height, cm	164.7±8.6
Weight, kg	64.9±11.8
Side, *n* (%)	
Left	80 (32.5)
Right	101 (41.1)
Bilateral	19 (7.7)
Kidney transplantation	46 (18.7)
Stricture location, *n* (%)	
Proximal	119 (44.9)
Middle	30 (11.3)
Distal	51 (19.2)
Ureterovesical anastomosis	65 (24.5)
Length of ureteral stricture, cm	3.4±3.0
Etiology of ureteral stricture, *n* (%)	
Following urinary stones surgery	77 (29.1)
Following urinary cancer	14 (5.3)
Following abdominal and pelvic surgery or radiotherapy	20 (7.5)
Following ureteroplasty	108 (40.8)
Following kidney transplantation	46 (17.4)

Procedure-related details were summarized in Table 2. The study included 246 patients with ureteral strictures, which were classified as common (*n*=92), recurrent (*n*=108), and postkidney transplantation (*n*=46) ureteral strictures. The study yielded an overall success rate of 71.7%. Success rates for common ureteral strictures, recurrent ureteral strictures, and ureteral strictures after kidney transplantation were 73.0, 67.6, and 78.2%, respectively. The mean operative time for patients with ureteral strictures after kidney transplantation (74.3±33.0 min) was longer compared to those with common ureteral strictures (68.7±33.7 min) and recurrent ureteroplasty (70.6±35.3 min). The average hospital stay time was 7.1 days, with a corresponding mean hospital cost of $ 9993.3. Eleven patients (4.2%) experienced MUS implantation failure and required drainage via a nephrostomy tube. Out of the 53 MUS that migrated, 33 were managed by adjusting the MUS position via ureteroscopy, while 20 required replacement with a new MUS. Eleven MUS were removed and exchanged due to MUS occlusion (six cases) or encrustation (five cases). Some patients experienced complications related to the MUS placement. Intermittent hematuria occurred in 21 (7.9%) patients, while 17 (6.4%) patients developed urinary tract infection. Eighteen (6.8%) patients experienced pain and discomfort, which resolved spontaneously. Fourteen (5.3%) patients experienced lower urinary tract symptoms. There were no stent-related deaths during the follow-up period.

**Table 2:** Summary of surgery details.

Variable	Common ureteral stricture	Recurrent stricture after ureteroplasty	Ureteral stricture after kidney transplantation	Total
Number of patients, *n*	92	108	46	246
Number of renal units, n	111	108	46	265
Release of obstruction, *n* (%)	106 (95.5)	104 (96.3)	44 (95.7)	254 (95.8)
Operative time, min	68.7±33.7	70.6±35.3	74.3±33.0	70.6±34.2
Hospital stay time, day	6.8±3.5	7.3±4.1	7.0±2.8	7.1±3.6
Total cost, $	9324.8±2328.8	10120.4±3716.1	11031.9±4579.7	9993.3±3503.6
Follow-up success rate, *n* (%)	81 (73.0)	73 (67.6)	36 (78.2)	190 (71.7)
Reasons for failure of surgery, *n* (%)				
Stent implantation failure	5 (4.5)	4 (3.7)	2 (4.3)	11 (4.2)
Stent migration	20 (18.0)	28 (25.9)	5 (10.9)	53 (20.0)
Stent occlusion	3 (2.7)	1 (0.9)	2 (4.3)	6 (2.3)
Stent encrustation	2 (1.8)	2 (1.9)	1 (2.2)	5 (1.9)
Symptoms with stents, *n* (%)				
Pain	7 (6.3)	6 (5.6)	5 (10.9)	18 (6.8)
Urinary tract infection	6 (5.4)	4 (3.7)	7 (15.2)	17 (6.4)
Hematuria	9 (8.1)	6 (5.6)	6 (13.0)	21 (7.9)
Lower urinary tract symptoms	5 (4.5)	5 (4.6)	4 (8.7)	14 (5.3)

The 2 years follow-up results are presented in Table 3, which shows a significant decrease in the volume of hydronephrosis (68.9±96.1 vs. 32.1±48.8 cm^3^, *P*<0.001), blood creatinine levels (103.7±49.8 vs. 94.4±47.5 μmol/l, *P*=0.004), and urea nitrogen levels (6.7±7.2 vs. 5.1±2.4 mmol/l, *P*=0.001) after a follow-up of 2 years.

**Table 3:** Summary of 2 years follow-up results.

	Preoperation				Follow-up results							
Variable	Common ureteral stricture	Recurrent stricture after ureteroplasty	Ureteral stricture after kidney transplantation	All	Common ureteral stricture	*P*	Recurrent stricture after ureteroplasty	*P*	Ureteral stricture after kidney transplantation	*P*	All	*P*
Hydronephrosis volume/mm^3^	62.4±83.0	66.6±94.1	89.0±125.3	68.9±96.1	36.7±50.4	0.0001	30.7±50.5	*P*<0.001	24.4±40.0	0.001	32.1±48.8	*P*<0.001
Creatinine (μmol/l)	96.3±47.8	108.3±54.2	107.9±41.5	103.7±49.8	95.8±51.5	0.925	95.9±47.8	0.033	88.2±37.8	*P*<0.001	94.4±47.5	0.004
Urea nitrogen (mmol/l)	5.9±2.3	7.0±7.9	7.5±11.0	6.7±7.2	5.4±2.7	0.050	5.1±2.3	0.014	4.6±1.6	0.078	5.1±2.4	0.001

## Discussion

Ureteral strictures are a common urological problem that can lead to impaired renal function and mortality if left untreated^2,12^. In addition, recurrent ureteral strictures after ureteroplasty or kidney transplantation are a challenging clinical problem that require multiple rounds of treatment^3,4,13^. The use of MUS has emerged as a viable treatment option for various types of ureteral strictures, with several advantages over traditional treatment modalities such as open surgery and plastic stents^8,14^. Several types of MUS are currently available, including Memokath, Resonance, Uventa, and Allium stents. These stents have been developed with specific design features and materials to meet the diverse clinical needs of patients with ureteral strictures. The Memokath stent is made of nitinol, offering shape memory properties and the ability to conform to the ureter’s natural shape while maintaining patency^15^. The resonance stent offers a full-length closed metal coil, providing radial force and excellent flexibility for optimal adaptation to the ureteral anatomy^16^. The Uventa stent is a unique ureteral stent that incorporates an inner mesh-polytetrafluoroethylene membrane and an outer mesh designed specifically to inhibit tissue ingrowth and minimize the occurrence of urothelial hyperplasia^17^. The Allium stent features a covered design with a biostable polymer layer, reducing tissue ingrowth and minimizing encrustation risks^7^. Besides, the Allium stent is easily endoscopic removal because its special unraveling feature even after a long indwelling period^7^.

Therefore, in our study, we specifically utilized the Allium stent. These metal stents have been extensively studied and have shown promising results in terms of their ability to maintain ureteral patency, provide adequate drainage, and improve patient outcomes.

In the context of our study, we have observed an overall success rate of 71.7%. Specifically, the success rates for common ureteral strictures, recurrent ureteral strictures, and ureteral strictures after kidney transplantation were found to be 73.0, 67.6, and 78.2% respectively. It is crucial to acknowledge that the success rate of these interventions is influenced by multiple factors. These include the underlying cause of the stricture, local anatomic factors specific to the ureteral stricture, and the surgical technique employed during the procedure. Notably, the length of the ureter tends to be relatively short after kidney transplantation. In this particular instance, by utilizing a 10 cm MUS, we were able to adequately support the entire length of the ureter. Consequently, the migration rate of the MUS in these cases were relatively low. On the other hand, for recurrent ureteral strictures following ureteroplasty, the presence of varying ureter diameters posed a challenge and increased the migration rate of the MUS. Among the 53 cases of migrated MUS, 33 were successfully managed by adjusting the position of the MUS through ureteroscopy, while 20 cases required replacement with a new MUS. Our findings indicate that certain surgical techniques can help minimize the occurrence of stent migration. For instance, stent implantation following adequate balloon dilation and the use of tandem stents for lengthy ureteral strictures have shown promising results in reducing migration rate. A total of 20 MUS that had migrated, 6 MUS that had become occluded, and 5 MUS that had become encrusted were removed and replaced with new MUS. The significant decrease in hydronephrosis volume, blood creatinine levels, and urea nitrogen levels observed after a follow-up of 2 years suggests that MUS can provide effective relief of ureteral obstruction and improve renal function in patients with ureteral strictures.

In comparison to double J tubes, Allium MUS have demonstrated a higher success rate and longer drainage time. Our surgical success rate using MUS was 71.7% over a 2-year follow-up period. In contrast, Chen *et al*.^18^ found that the success rates for double J tubes at 6 months and 1 year after the operation were 83.8 and 40.0%, respectively. Additionally, the overall complication rate of metal stents was lower than that of ordinary ones, with rates of 36.7 versus 63.6%, respectively^18^. Allium MUS are widely utilized in various medical centers. Guandalino *et al*.^19^ reported a success rate of 52.8% after a mean follow-up of 7.1 months, which is slightly lower than our success rate. It is important to recognize that the success rates of various centers is impacted by several factors, which encompass the underlying cause of the stricture and specific local anatomic factors related to the ureteral stricture. These variables play a significant role in determining the overall outcome and should be taken into consideration when evaluating the success rates of different centers.

Complications related to MUS placement were observed in our patients, with hematuria and low back pain being the most common. These findings are consistent with previous studies that have reported similar complication rates with MUS^20,21^. However, the overall complication rate observed in our study was relatively low, suggesting that MUS are a safe treatment option for ureteral strictures.

The longer operative time observed in patients with ureteral strictures after kidney transplantation is not unexpected, given the technical challenges associated with the surgical management of this patient population. However, the overall mean operative time of 74.3 min observed in our study compares favorably with previously reported operative times for MUS placement^22,23^.

This study is limited by its single-center setting. The results may not be generalizable to other patient populations or treatment centers. Additionally, the study is limited by the lack of a control group for comparison of outcomes. Larger, multicenter studies with longer follow-up periods are needed to confirm our findings and further evaluate the safety and efficacy of MUS in the management of ureteral strictures.

## Conclusions

Our study demonstrates that MUS are a safe and effective treatment option for ureteral strictures, with a high success rate and durable patency. The use of MUS can provide effective relief of ureteral obstruction and improve renal function in patients with ureteral strictures.

## Ethical approval

Ethical approval was approved by the Ethics Approval Committee of West China Hospital, and the registered number was 2019-009.

## Sources of funding

This study was supported by the new clinical technology in West China Hospital of Sichuan University (grant no. 20HXJS002); Postdoctoral Fund of West China Hospital of Sichuan University (grant no. 2023HXBH041); Natural Science Foundation of Sichuan Province (grant no. 2023NSFSC1532). Project of Science and Technology Department of Sichuan Province (2022NSFSC0712).

## Author contribution

X.G.: Writing original draft; X.D.: conceptualization, methodology; L.P.: investigation; G.C.: software; W.W.: reviewing; J.C.: data curation; X.W.: supervision.

## Conflicts of interest disclosure

The authors declared that they had no competing interest related to this work.

## Research registration unique identifying number (UIN)

1. Name of the registry: Research Registry.
2. Unique Identifying number or registration ID: researchregistry7266.
3. Hyperlink to your specific registration (must be publicly accessible and will be checked): https://www.researchregistry.com/browse-theregistry#home/.

## Guarantor

Xin Wei.

## Data availability statement

Research data used in the study will be available on request.

## Provenance and peer review

Not commissioned, externally peer-reviewed.
